# TeV/m catapult acceleration of electrons in graphene layers

**DOI:** 10.1038/s41598-023-28617-w

**Published:** 2023-01-24

**Authors:** Cristian Bonţoiu, Öznur Apsimon, Egidijus Kukstas, Volodymyr Rodin, Monika Yadav, Carsten Welsch, Javier Resta-López, Alexandre Bonatto, Guoxing Xia

**Affiliations:** 1 Department of Physics, University of Liverpool, Oxford Street, Liverpool, L69 7ZE UK; 2 Cockroft Institute, Keckwick Lane, Daresbury, Warrington, WA4 4AD UK; 3 ICMUV, Instituto de Ciencia de los Materiales, Universidad de Valencia, 46071 Valencia, Spain; 4 Graduate Program in Information Technology and Healthcare Management, and the Beam Physics Group, Federal University of Health Sciences of Porto Alegre, Porto Alegre, RS 90050-170 Brazil; 5 Department of Physics and Astronomy, University of Manchester, Oxford Road, Manchester, M13 9PL UK

**Keywords:** Laser-produced plasmas, Nanophotonics and plasmonics

## Abstract

Recent nanotechnology advances enable fabrication of layered structures with controllable inter-layer gap, giving the ultra-violet (UV) lasers access to solid-state plasmas which can be used as medium for electron acceleration. By using a linearly polarized 3 fs-long laser pulse of 100 nm wavelength and 10$^{21}$ W/cm$^2$ peak intensity, we show numerically that electron bunches can be accelerated along a stack of ionized graphene layers. Particle-In-Cell (PIC) simulations reveal a new self-injection mechanism based on edge plasma oscillations, whose amplitude depends on the distance between the graphene layers. Nanometre-size electron ribbons are electrostatically catapulted into buckets of longitudinal electric fields in less than 1 fs, as opposed to the slow electrostatic pull, common to laser wakefield acceleration (LWFA) schemes in gas-plasma. Acceleration then proceeds in the blowout regime at a gradient of 4.79 TeV/m yielding a 0.4 fs-long bunch with a total charge in excess of 2.5 pC and an average energy of 6.94 MeV, after travelling through a graphene target as short as 1.5 $\upmu$m. These parameters are unprecedented within the LWFA research area and, if confirmed experimentally, may have an impact on fundamental femtosecond research.

## Introduction

Unlike LWFA in gases, which can be achieved with laser pulses in the infra-red (IR) range, at peak intensities of $10^{18}$–$10^{19}$ W/cm$^2$^1^, the equivalent mechanism in solid-state plasmas requires $10^{20}$–$10^{21}$ W/cm$^2$ laser pulses in the UV range. Motivated by recent developments in laser science such as thin film compression^2^ and relativistic surface compression^3^, single-cycle IR laser pulses were also considered as drivers for LWFA in nanotubes^4^. However, neither numerical nor theoretical studies have been published on the possibility to accelerate electrons using graphene layered targets in combination with UV laser pulses. Pure graphene layers contain $1.14\times 10^{23}$ atoms/cm$^3$, that is 4–5 orders of magnitude more than the pressurized gases commonly used for LWFA^5^. Graphene targets can be grown in the form of many 2D layers of Carbon atoms stacked together^6^. Each layer is 0.34 nm thick and, when fully ionized delivers a plasma density of $6.84 \times 10^{23}$ cm$^{-3}$. Ionization with a sufficiently intense laser pulse, ensures that electrons leave the layers to form a virtually homogeneous cloud, with a density of $\sim$ 10$^{22}$ cm$^{-3}$ through the Brunel non-resonant mechanism^7,8^. More exactly, for a graphene target made of 60 layers stacked with an inter-layer gap of 20 nm, as used in this work, the effective electron plasma density at complete ionization is $n_e$ = $1.16 \times 10 ^{22}$ cm$^{-3}$. With the electron mass $m_e$ and charge *e*, and vacuum electric permittivity $\epsilon _0$, the plasma angular frequency defined as

1 $$  \omega _p = \sqrt{ \frac{n_e e^2}{m_e \varepsilon _0}}, $$

can be used to assess the viability of a laser pulse of 100 nm wavelength. Key plasma and laser parameters are listed in Table  1, showing that the interaction falls in the overdense regime ($\omega _p > \omega _0$) for the layer plasma and in the underdense regime ($\omega _p < \omega _0$) for the cloud plasma, where $\omega _p$ and $\omega _0$ are the angular frequencies for the plasma and laser, respectively.

**Table 1:** Layer and cloud plasma versus laser parameters.

Parameter	Layer	Cloud	Laser	Unit
Plasma density, $n_e$	6.84$\, \times \,$10$^{23}$	1.16$\, \times \,$10$^{22}$	–	cm$^{-3}$
Angular frequency	46.66	6.08	18.84	$\, \times \,$ 10$^3$ rad-THz
Wavelength	40	310	100	nm

**Figure 1:**
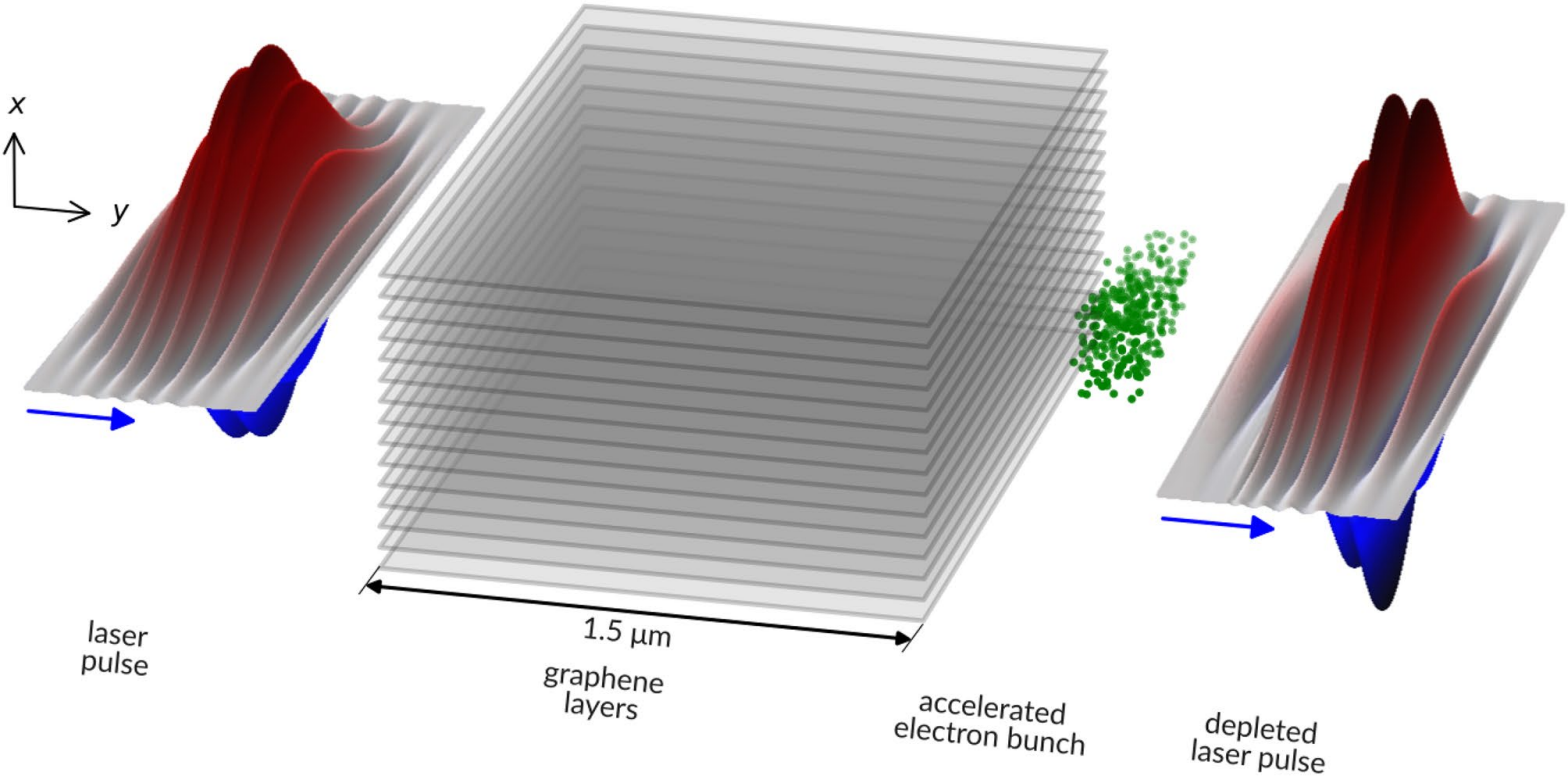
Overview of the catapult electron acceleration scheme in graphene layers. Moving from left to right, as indicated by the blue arrows, a single 3 fs-long laser pulse of 100 nm wavelength and 10$^{21}$ W/cm$^2$ peak intensity, ionizes a 1.5 $\upmu$m-long (y) and 1.2 $\upmu$m-thick (x) stack of graphene layers. The interaction results in self-injected electrons being accelerated to $\simeq$ 7 MeV. The image is at scale, and for better visibility, only 15 out of 60 graphene layers are shown. The simulated normalized transverse electric field ($E_x$) is shown as a surface colour plot for the same laser pulse before entering the target (left) and after leaving the target (right). This work contains 2D PIC simulations carried out in the *yx*-plane indicated in the image.

Through PIC simulations carried out with PIConGPU^9^, we show that using a laser pulse of 100 nm wavelength, electron self-injection is possible from the edge of the multilayer graphene plasma, provided that the laser pulse is sufficiently intense and energetic. Accelerated electron bunches can be extracted at the other edge of the target, following the interaction scheme shown in Fig. 1.

As opposed to the LWFA schemes in gases, here self-injection is due to the short ($\sim$ 0.5 fs) burst of a longitudinal electric force at the left edge of the target. In addition, all charge is injected at once, as a projectile, and remains virtually constant thereafter. These two observations motivate us to name the injection and acceleration scheme “catapult”.

## Results

The interaction is modelled using a linearly polarized Gaussian laser pulse whose parameters are shown in Table  2. The pulse moves along the *y*-axis while its electric field oscillates in the simulation plane *yx*. The blowout regime^10^ occurs if the width of the target (here 1.2 $\upmu$m) is larger than the pulse length (here 0.9 $\upmu$m). This is due to the coupled oscillatory motion of the ionized electrons, described below.

**Table 2:** Laser parameters.

Quantity	Value	Unit
Wavelength, $\lambda$	100	nm
Period, *T*	0.334	fs
Peak intensity, $I_0$	10$^{21}$	W/cm$^2$
Spot size (FWHM), $w_0$	0.4	$\upmu$m
Focal point, $y_f$	0.25	$\upmu$m
Pulse energy, *E*	8	mJ
Pulse length (9 cycles), $\Delta t$	3	fs
Potential vector, $a_0$	2.7	–

**Figure 2:**
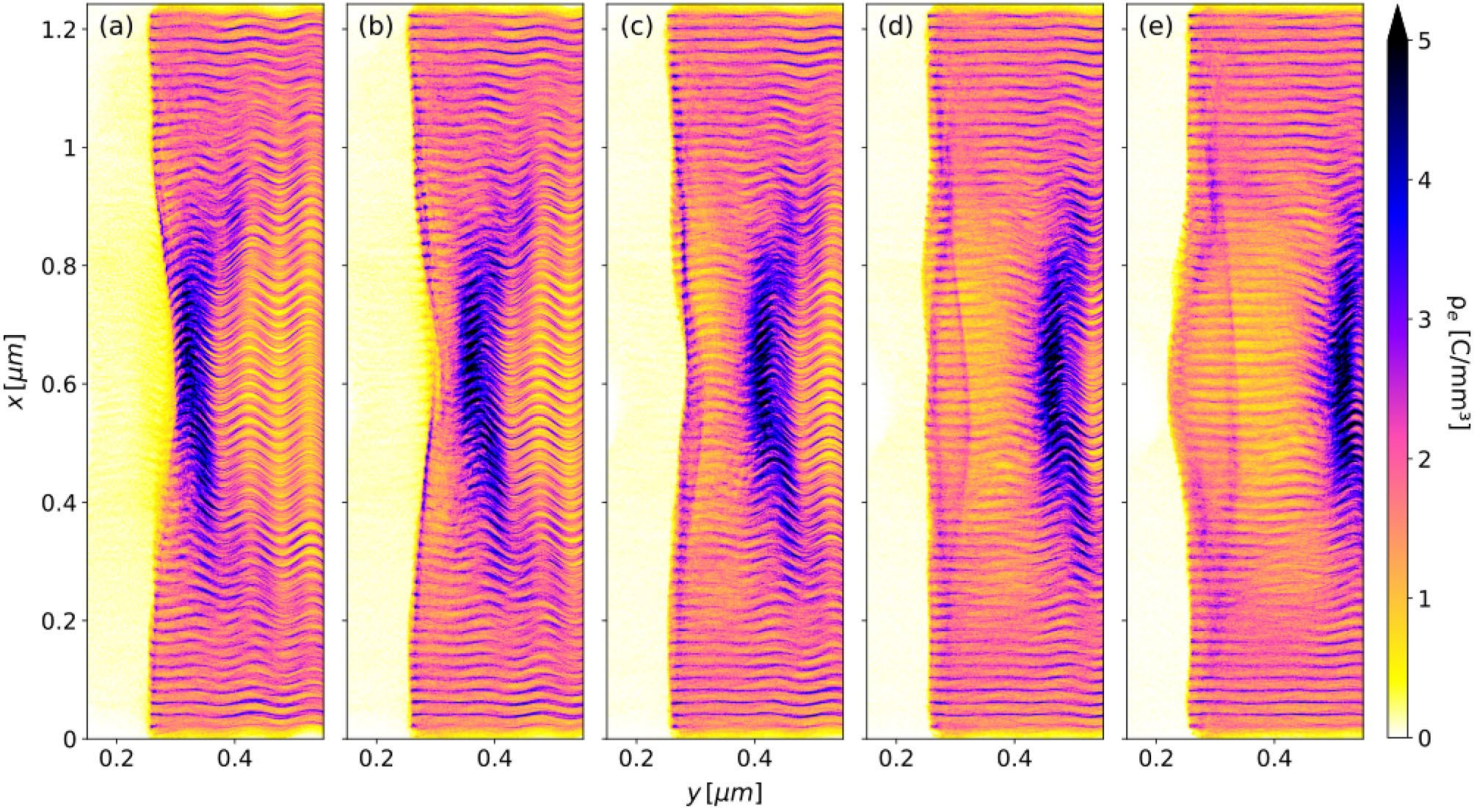
The catapult injection mechanism, shown as electron charge density at: (**a**) *t*/*T* = 10.5, (**b**) *t*/*T* = 11, (**c**) *t*/*T* = 11.5, (**d**) *t*/*T* = 12 and (**e**) *t*/*T* = 12.5.

As the first laser cycle hits and ionizes the layers at the left edge, electrons are repelled transversely upwards and downwards by the alternating laser field $E_x$. With the following laser cycles, their transverse motion grows in amplitude but there is also a concurrent longitudinal oscillation along the layers of carbon ions left at rest and electrically unbalanced. While executing these combined 2D oscillations most of the electrons leave the laser pulse region, being initially confined near the transverse extremities of the target ($x<$ 0.2 $\upmu$m and $x>$ 1 $\upmu$m). With the laser pulse advancing along the target, these electrons then collapse towards the left edge of the target, by this point nearly void of electrons. One of the outcomes is the appearance of a thick wall of electrons, just behind the laser pulse, as shown in Fig. 2a, but another one, key to this work, is that while most of the wall follows the laser pulse, being continuously replenished, its left extremities are attracted leftwards by the ions, initiating a damped oscillation which lasts for about 36 laser cycles. This split between the electrons in the wall and those moving leftwards, gradually builds up a bubble of ions. Furthermore, from the electrons moving leftwards, a $\sim$ 10 nm-thick ribbon is catapulted into the left half of the bubble due to the favourable longitudinal electric field $E_y$ just being formed. This behaviour, shown in Fig. 2b–e, is of paramount importance to the injection and acceleration process. Electrons oscillation about the target end is essentially a spill-out nanoplasmonic effect^11^ studied within the realm of nanoelectronics, but never considered as a mechanism to inject electrons into a laser-plasma accelerator. The efficiency of the catapult process ultimately depends on the ratio between the laser wavelength and the inter-layers gap. In this work the wavelength/gap ratio is 5. While for wider gaps laser propagation is longer, which is an expected behaviour, given the lower effective plasma density, electrostatic forces at the left edge of the target are weaker and less charge is available for injection. It is worth mentioning that the catapult process is a femtosecond electron injection scheme, with all charge injected at once as opposed to the LWFA in gases, where electrons are gradually dragged into the bubble within a few ps^12^.

**Figure 3:**
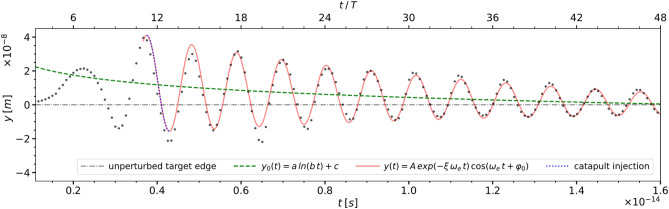
The electron plasma at the left edge of the multilayer graphene target executes a damped longitudinal oscillation along the *y* axis with a mean $y_0$ (dashed green line), which converges logarithmically to the initial plasma edge boundary (grey dash-dotted line). The relative elongation (black dots) is fitted with a damped oscillator model (red solid line). Injection starts at *t*/*T* = 11.25 when the plasma edge recedes leftwards and ends by *t*/*T* = 12.84 when the plasma edges moves again rightwards. In Fig. 2, the sub-figures (c), (d) and (e) show the electron charge density during injection.

**Figure 4:**
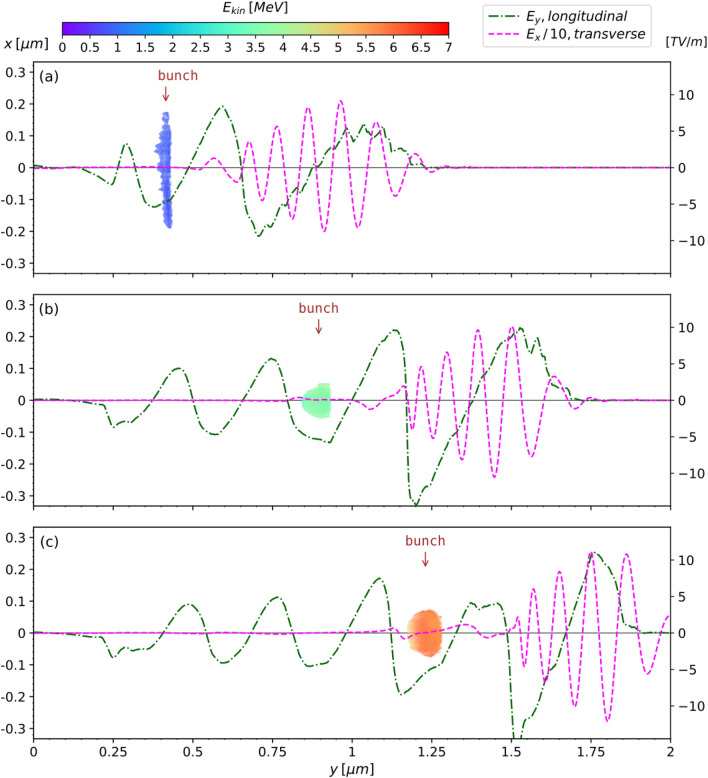
On-axis longitudinal electric field $E_y$ and transverse electric field $E_x$ arising mostly from the laser pulse, overlapped with the electron bunch at: (**a**) *t*/*T* = 13.7, (**b**) *t*/*T* = 19 and (**c**) *t*/*T* = 22.5.

The longitudinal oscillations at left edge of the target, which are shown in Fig. 3, can be modelled as a damped oscillator:

2 $$\begin{aligned}  y(t) = A\,exp(-\xi \,\omega _e\,t)\,cos(\omega _e\,t + \varphi _0) \end{aligned}$$

whose mean follows a logarithmic curve:

3 $$  y_0(t) = a\,ln(b\,t) + c $$

where *t*, denotes time. The parameters of the logarithmic curve $y_0$ are: $a = -8.17\times 10^{-9}$ m, $b = 2.59 \times 10 ^{-7}$ s$^{-1}$ and $c = -3.83 \times 10 ^{-7}$ m, while the parameters of the damped oscillator are $A = 45 \times 10 ^{-9}$ m, $\omega _e$ = 2$\,\pi \,c$/$\lambda _e$, $\lambda _e = 320.9 \times 10 ^{-9}$ m, $\xi$ = $2.05 \times 10 ^{-2}$ rad$^{-1}$, and $\varphi _0$ = 0.98$\,\pi$, with *t* in s. Both $\omega _e$ and $\lambda _e$ should be compared with the corresponding *Cloud* values listed in Table 1.

with a smaller angular frequency ($\omega _e$) than that of the bulk plasma ($\omega _p$). The quality factor of the oscillator is

4 $$\begin{aligned}  Q = \frac{\omega _p}{\omega _p - \omega _e} = 29.2, \end{aligned}$$

which confirms that the oscillation is under-damped. In this scheme electrons are injected not only in the first bubble but also in subsequent bubbles, although with decreasing overall charge. With further optimization, the catapult process could become a unique scheme of obtaining trains of electron bunches separated by a few fs. Within the bubble, the transverse electric field components $E_x$, as well as the azimuthal magnetic field $B_z$ created by the electrons moving inside and leaking from the laser pulse, are simultaneously focusing the electron ribbon into a compact bunch. The bunch is focused from an initial transverse FWHM size of 265 nm shown in Fig. 4a, to a minimum transverse FWHM size of 65 nm shown in Fig. 4b, during about 5 laser cycles. Thereafter, the bunch is defocused, as shown in Fig. 4c, due to the growing space charge forces which oppose the decreasing focusing forces. The catapult injection delivers relativistic electrons with $\beta \sim$ 0.90 and therefore, as it can be seen from Fig. 4 the bunch does not significantly slip out of phase while gaining energy. However, it flattens the accelerating field $E_y$ through beam loading^13^.

**Figure 5:**
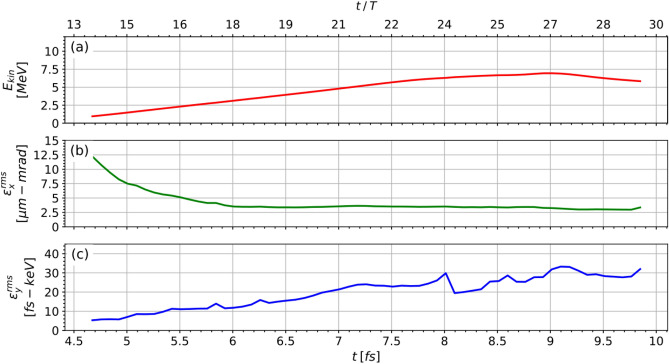
Evolution of key bunch parameters: (**a**) mean kinetic energy, (**b**) transverse emittance, (**c**) longitudinal emittance.

As the bunch is accelerated through the target, the rate of energy gain decreases. This is shown in Fig. 5a. The transverse emittance $\varepsilon _x$, shown in Fig. 5b, is damped within the first 3 laser cycles after injection and remains virtually constant afterwards without being affected by the dechanneling effects^14^. On the contrary, the longitudinal emittance $\varepsilon _y$, shown in Fig. 5c, grows steadily as the bunch elongates longitudinally.

**Figure 6:**
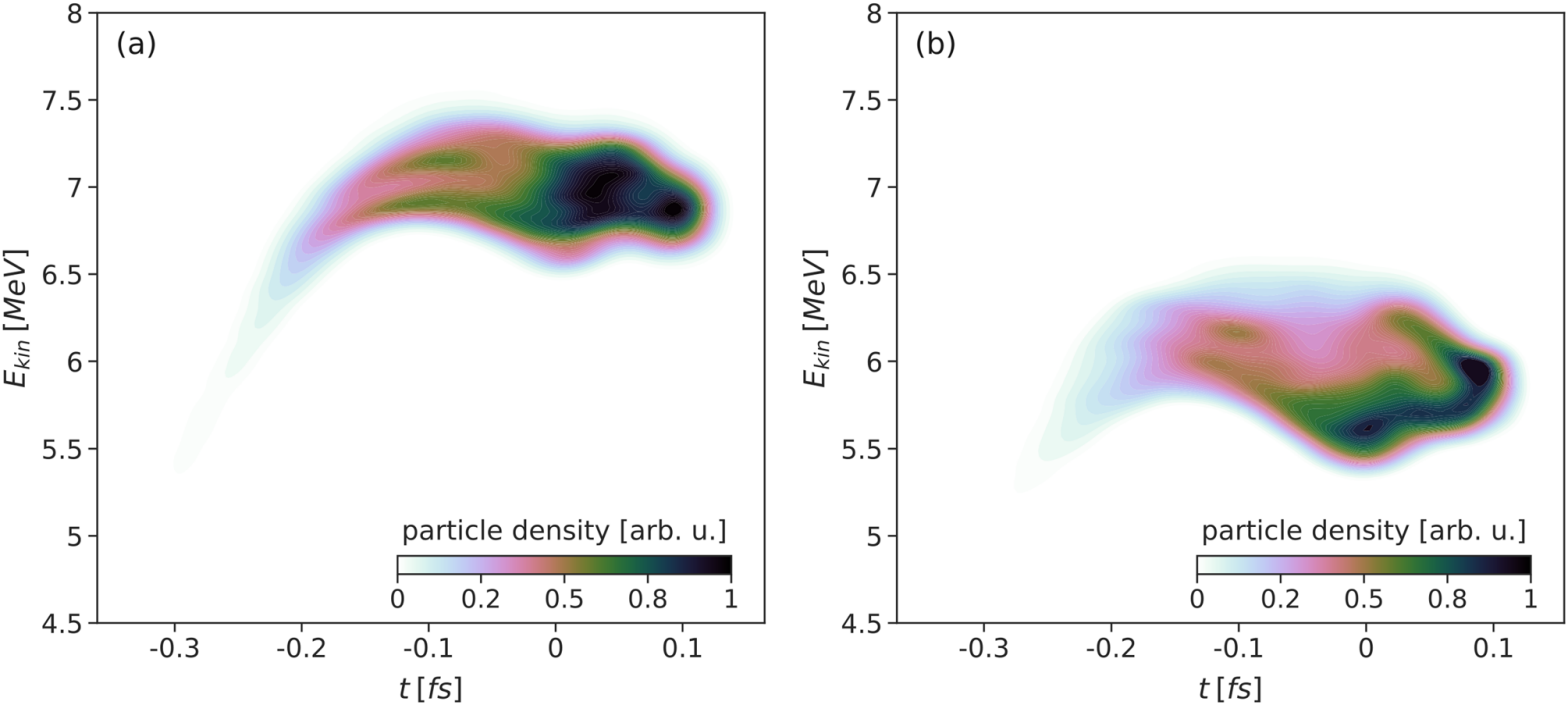
Longitudinal phase space: (**a**) before extraction, at *t*/*T* = 27, with the mean kinetic energy E$_{kin}$ = 6.94 MeV and FWHM energy spread $\Delta$E = 7.4$\%$ and (**b**) after extraction, at *t*/*T* = 30 with the mean kinetic energy E$_{kin}$ = 5.77 MeV and FWHM energy spread $\Delta$E = 12.3$\%$.

During the time in which the laser pulse sweeps over the right target edge, it removes a large amount of background electrons, creating a strong longitudinal electric field of opposite sign as compared to the one previously sustained in the second half of the wakefield bubble, and used by the bunch to gain energy. As it can be seen from two snapshots of the longitudinal phase space, shown in Fig. 6, the bunch loses some energy when traversing this field and leaving the target.

**Figure 7:**
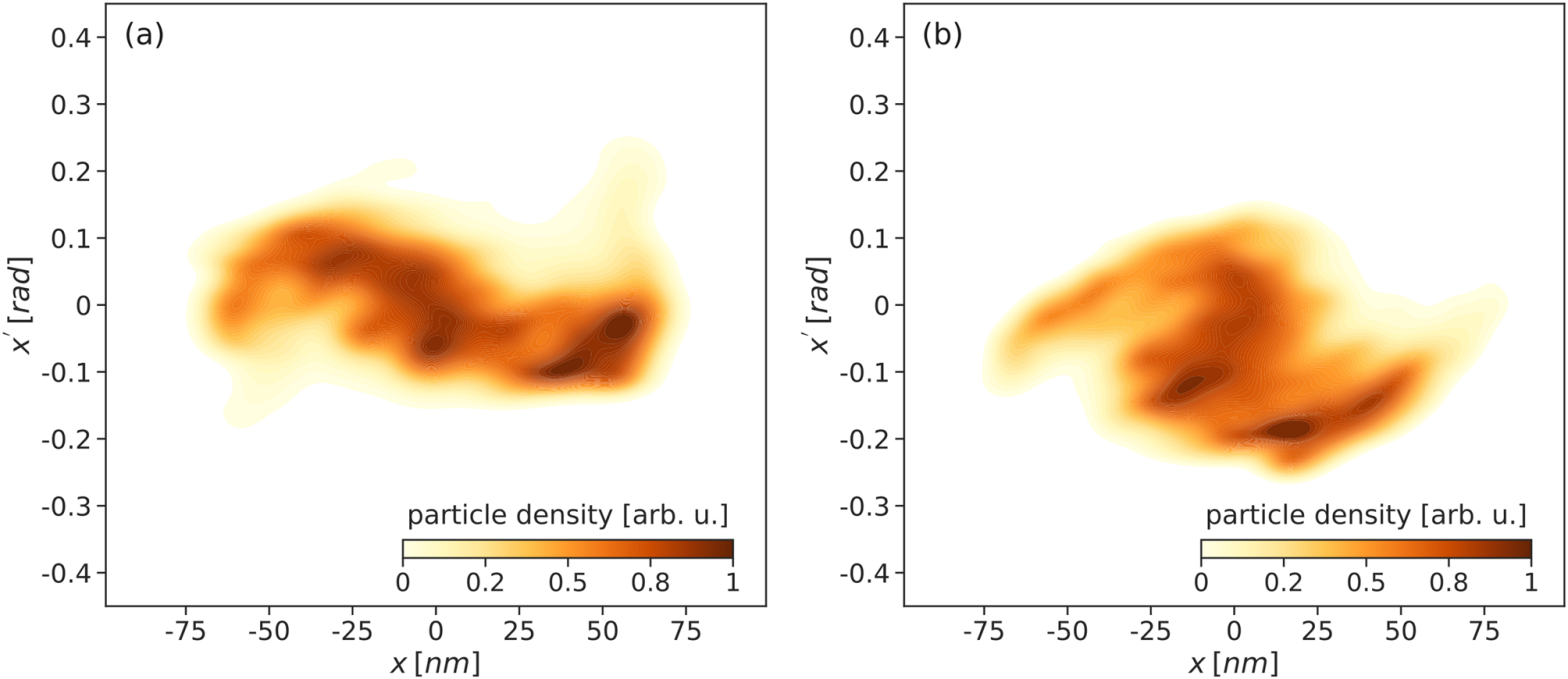
Transverse phase space: (**a**) before extraction, at *t*/*T* = 27, and (**b**) after extraction, at *t*/*T* = 30. There are several lumps as a consequence of the transverse focusing caused by the layers of carbon ions. The bunch is diverging under the action of its own space charge, as the transverse focusing provided by the wakefield bubble disappears.

Similarly, the transverse phase space at the same two snapshots is shown in Fig. 7 revealing that the electron bunch diverges immediately after leaving the target.

**Figure 8:**
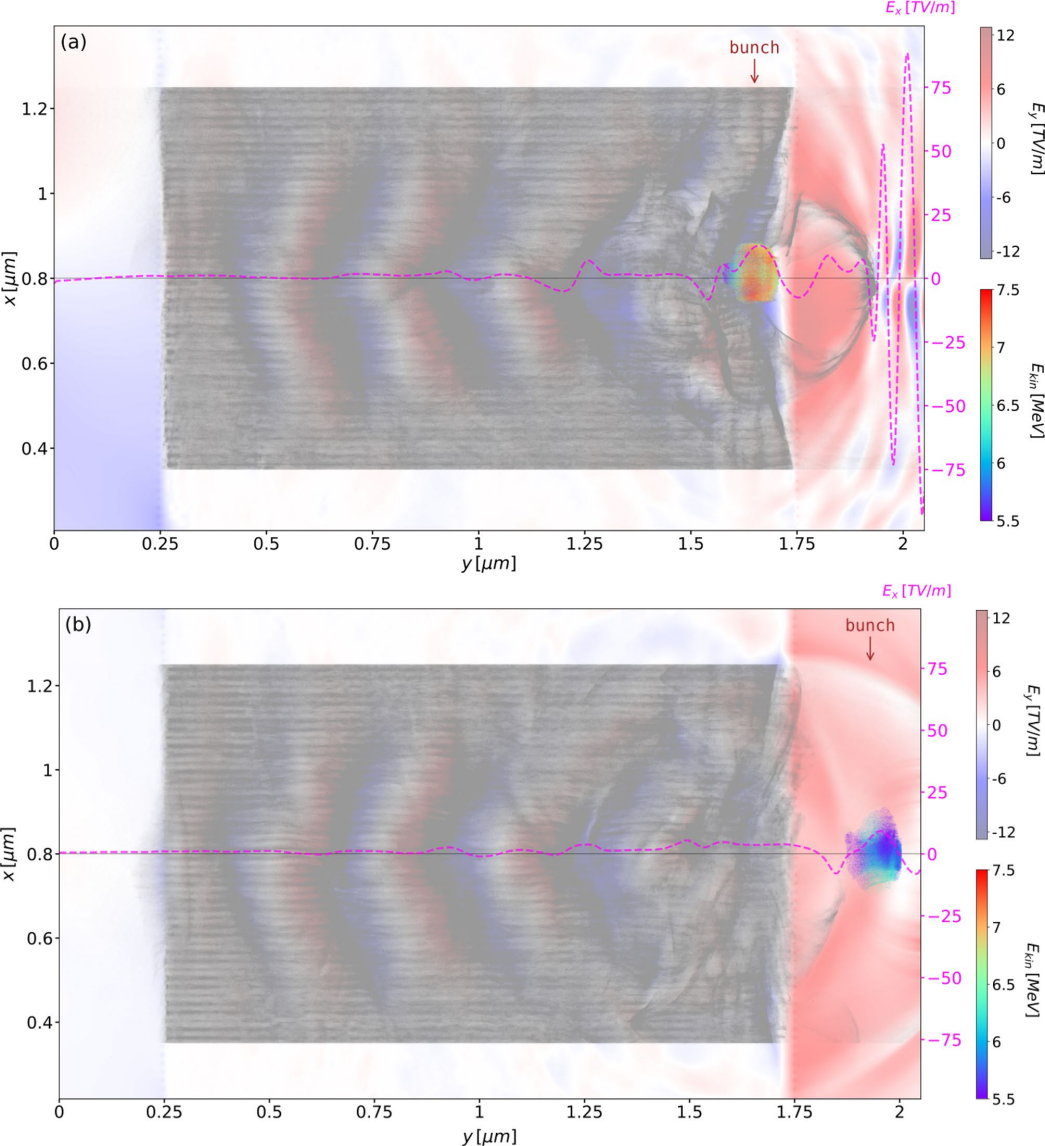
Electron macroparticles shown at: (**a**) *t*/*T* = 27 and (**b**) *t*/*T* = 30, for the target (grey dots) and for the accelerated bunch (rainbow-coloured dots with the colour map representing the kinetic energy). The laser pulse is shown through the on-axis transverse electric field $E_x$ (dashed purple line). It depletes the layers of electrons, building up regions of negative and positive longitudinal electric field (red-blue colour map).

Figures  6a,b and 7a,b in the phase space correspond respectively to the bunch shown in Fig. 8a,b in the real space, in the following section.

## Discussion

As the bunch approaches the right end of the multilayer graphene target, it encounters waves of backward travelling electrons, due the longitudinal oscillations driven by the laser pulse. The oscillations remove electrons from the right edges of the graphene layers, creating a favourable rightwards acceleration gradient for the electrons contained in the first wall behind the laser pulse. As shown in Fig. 8, a halo of background electrons is ejected and expands radially.

This phenomenon precedes ion Coulomb explosion^15^ used in target normal sheath acceleration schemes^16^ with IR lasers. It is worth mentioning that the catapult self-injection and acceleration was demonstrated as high-energy (mJ) collective phenomenon, without accounting for the appearance of the plasmons in the solid-state lattice, as considered elsewhere^17^. Finally, the performance is shown in Table 3, in which the 3D bunch charge was obtained by scaling the 2D value up to the equivalent of the FWHM transverse laser spot size. Although the kinetic energy and bunch charge are smaller than those obtained in the most recent LWFA experiments^18^ by a factor of $10^{3}$ and 2 respectively, the acceleration gradient of the catapult scheme is larger by a factor of 10. Furthermore, the normalized rms transverse emittance defined as

5 $$  \varepsilon _x^n = \beta \gamma \varepsilon _x = 3.8\times 10^{-2}\;\text {mm-mrad}, $$

when divided by the bunch charge *q*, is smaller than in other LWFA schemes^19^ by a factor of 20.

**Table 3:** Parameters of the bunch before extraction (*t*/*T* = 27) and after extraction (*t*/*T* = 30).

Quantity	Value at		Unit
	*t*/*T* = 27	*t*/*T* = 30	
Kinetic energy, $E_{kin}$	6.94	5.77	MeV
Energy spread (FWHM), $\Delta E$	7.40	12.29	$\%$
Longitudinal rms emittance (unnormalized), $\varepsilon _y$	31.85	31.75	fs$\text {-}$keV
Transverse rms emittance (unnormalized), $\varepsilon _x$	$3.25\times 10 ^{-3}$	$3.11 \times 10 ^{-3}$	mm$\text {-}$mrad
Total charge (3D equivalent), *q*	2.55		pC
Transverse size (FWHM), $\Delta x$	103	65	nm
Transverse divergence (FWHM), $\Delta x'$	220	317	mrad
Longitudinal duration (FWHM), $\Delta t$	0.208	0.209	fs
Relativistic velocity factor, $\beta$	0.998	0.996	–
Relativistic Lorentz factor, $\gamma$	14.580	12.287	–

We have shown that multilayer graphene can sustain TV/m longitudinal electric fields. With the advent of UV laser sources^20–22^ and the development of Thin Film Compression techniques for UV lasers, following a similar approach used for IR lasers^2^, the catapult phenomenon described in this article offers a promising path towards the generation of sub-femtosecond-long electron bunches with a mean kinetic energy of several MeV. This shows exciting prospects for delivering the shortest electron bunches ever produced in the laboratory with excellent potential to advance ultra-fast electron diffraction techniques beyond current limits^23,24^. Another potential application is the generation of THz magnetic impulses with the current techniques aiming for time resolutions in the order of tens of fs^25^. Overall, this work demonstrates that laser-wakefield acceleration in solid state plasma can be achieved without the need of X-ray lasers as previously thought^26,27^, and therefore has the potential to direct current research on novel acceleration techniques towards using UV laser pulses and layered nanomaterials. Current techniques for growing high-quality graphene nanoribbons, such as Chemical Vapour Deposition, focus on applications related to electronics and spintronics^28,29^ which require atom size precision. From this point of view, the catapult scheme may work with larger tolerances since it is a collective phenomenon of the target. Graphene can withstand laser intensities up to 10$^{12}$ W/cm$^{-2}$, but as the catapult scheme presented here requires 10$^{21}$ W/cm$^{-2}$, the target is structurally damaged and cannot be reused. However, given the high inertia of the Carbon ions and because the electron bunch is formed, accelerated and extracted less the 2 fs behind the laser pulse maximum, the scheme is viable. The electron bunch exhibits the electrostatic pull of a virtually undamaged lattice of Carbon ions.

## Methods

**Figure 9:**
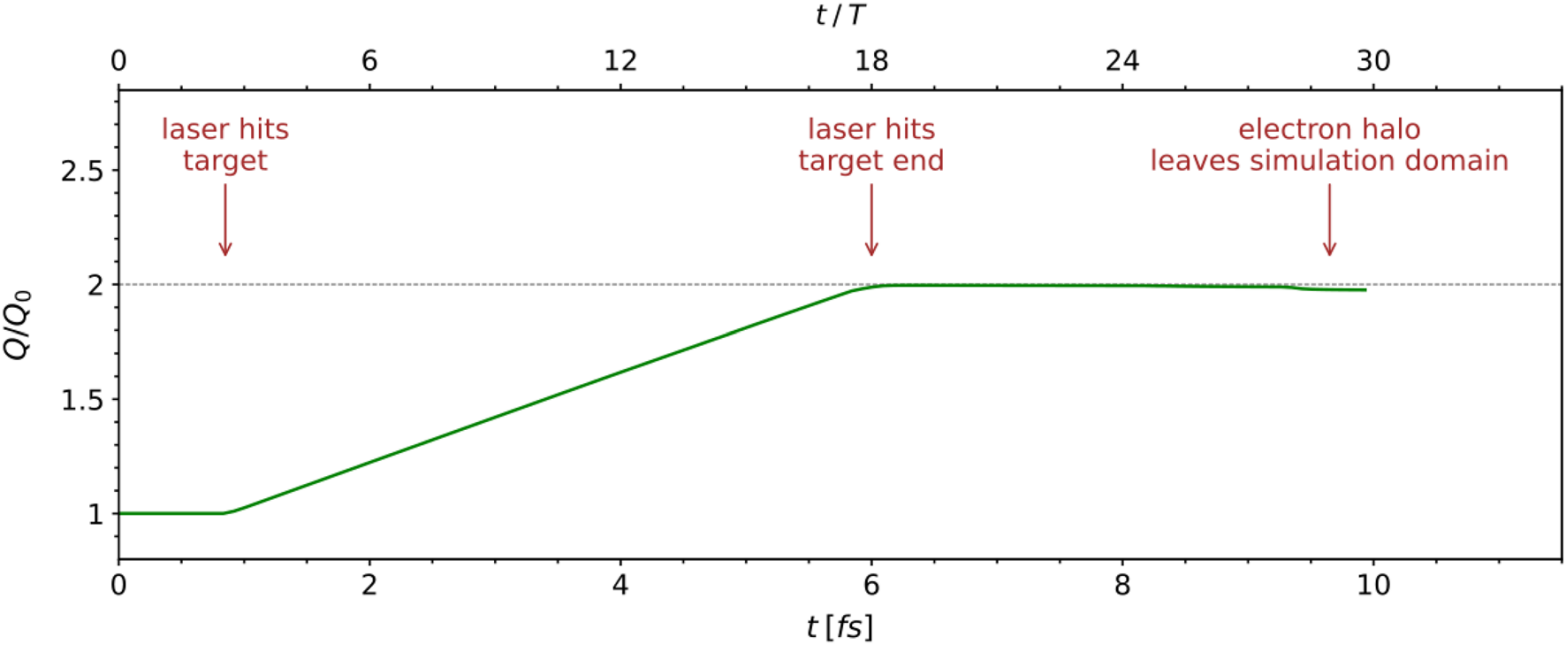
The maximum ratio between the total charge *Q* in the simulation domain at any given time and the total charge $Q_0$ at *t* = 0, proves that ionization is complete. There are 3 “free” electrons for each Carbon atom at *t* = 0 and twice as many for each Carbon atom when the laser pulse reaches the right edge of the target.

When compared with theoretical methods, PIC methods^30^ provide a complementary understanding of charged-particle dynamics due to their ability to include arbitrary target geometries and laser pulse envelopes in two or three dimensions. The simulations were performed in a box of 2 $\upmu$m $\times$ 1.6 $\upmu$m with a rectangular mesh cell of 0.135 nm $\times$ 0.135 nm, which corresponds to 2.51 cells per layer thickness, and 10 macroparticles per cell, as these were the limitations of the available hardware. The target length along the *y*-axis is set to 1.5 $\upmu$m as a realistic dimension of the graphene layers available in the near future and, to understand the edge effects, an empty region is considered around the target. Three ionization mechanisms were enabled: tunneling^31^, p. 277, barrier suppression^32,33^ and collision^34^. It is worth mentioning that unlike with the LWFA in low-Z gases, ionization through collision is significant. The PIConGPU code^9^ was chosen due to its capability to scale performance with the number of available graphics cards, but also due to the rich variety of technical features such as macroparticle initialization, ionization mechanisms, field solvers etc. A recent validation of PIConGPU^35^ was published in the context of acceleration driven in plasma by laser-accelerated electron beams. The code was also used to demonstrate generation of high-energy proton micro-bunches in mixed species gases^36^. Given the extremely short simulation time of 10 fs, we could not consider the case of a fully preionized plasma by a previous laser pulse, as it is usually done with IR LWFA simulations^36^. It must be noted that although the simulations are carried out in two dimensions namely *y* (longitudinal) and *x* (transverse), numerically the third dimension, along the *z*-axis (out-of-plane) is present as a single mesh cell, 0.135 nm deep, and this allows a meaningful retrieval of quantities such as electron charge density $\rho _e$ in C/m$^3$, out-of-plane magnetic field $B_z$, momentum $p_z$ etc, in what is called a 2D3V setup. PIConGPU is a gas-plasma code with the fields resolved on staggered Yee grid^37^ and the motion of the particles simulated by a Boris-type pusher^38^, which assumes unrestricted motion of both electrons and Carbon ions. Suppression of accelerating gradients in hydrogen plasmas due to the ion motion was previously discussed^39^. Here, although the electron to Carbon mass ratio is $\sim$ 10$^{-4}$, given the high laser intensity and virtually instant complete ionization inside the laser pulse, an evaluation of the Carbon ion displacement was carried out. There is virtually no displacement under the impact of $\simeq$ 70 TV/m laser electric field and at most 1–3 nm displacement as the simulation completes. However, by that time the accelerated electrons have left the target. This result supports previous similar findings that wakefields in solid-state nanomaterials remain virtually unaffected by the ion dynamics^27^. Concluding, the code can be safely applied to simulate electron laser-driven acceleration in a solid-state lattice. All simulations presented so far in this article were carried out using Carbon atoms in the 3rd ionization state (C$^{3+}$), to account for rather weaker first ionization potentials of graphene^40^ as compared with those of the Carbon atom. As the simulation progresses, a proof of complete ionization is shown in Fig.  9, where the ratio between total electron charge in the simulation domain at any given time *Q*(*t*) and that at the beginning of the simulation $Q_0(t = 0)$ is shown. At $t/T\,\simeq$ 3 the laser pulse hits the left edge of the layered graphene target and ionization starts through all three types listed above. By $t/T\,\simeq$ 18, the total electron charge in the system doubles and remains constant thereafter, as the laser pulse emerges at the right edge of the layered graphene target.

**Figure 10:**
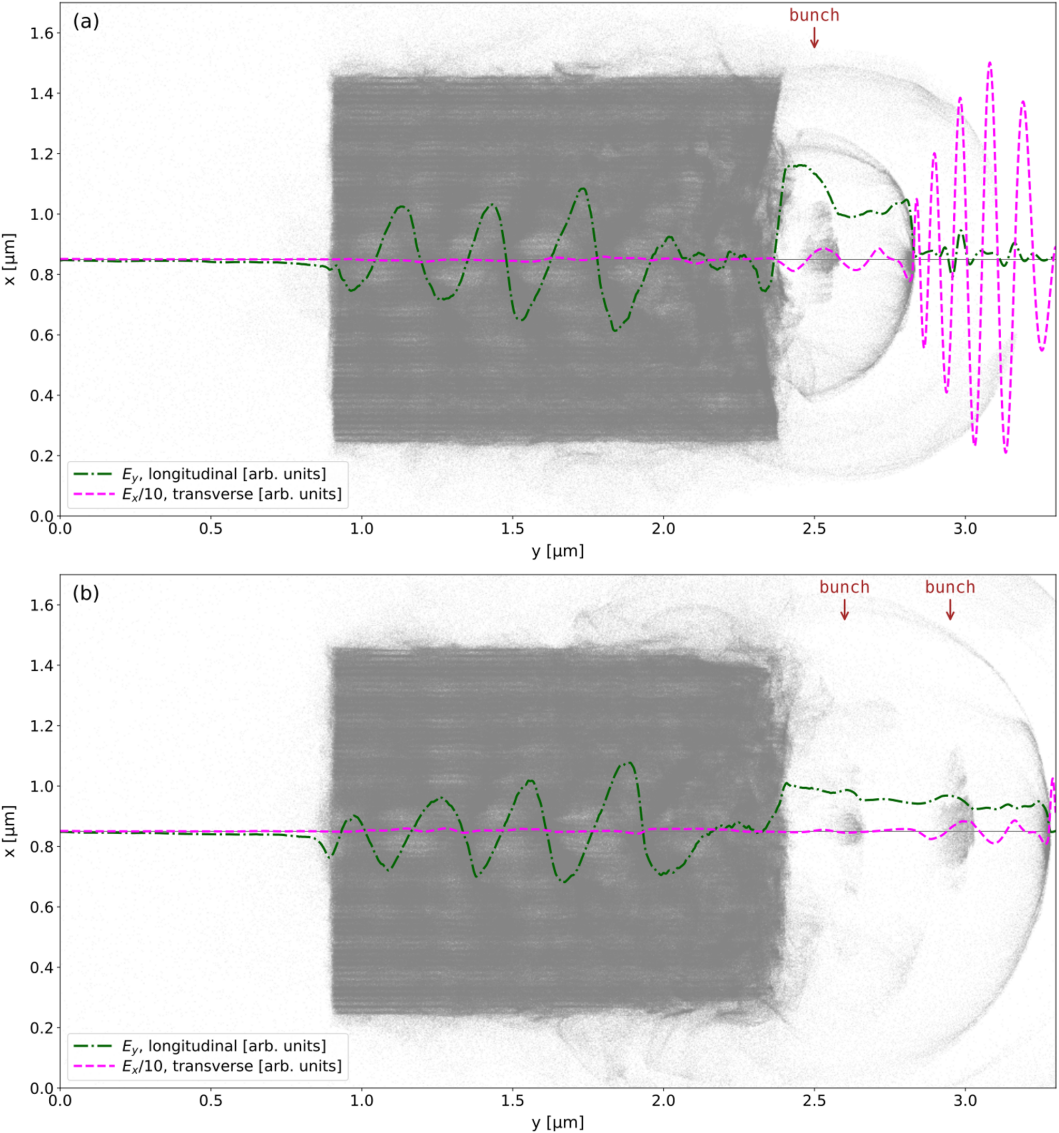
Extraction of the electron bunches in the wake of the laser pulse, shown by the dashed magenta line, using unionized Carbon atoms: (**a**) two halos are formed as the laser pulse leaves the target, with the first electron bunch extracted and (**b**) 4.5 laser cycles later, a second electron bunch of lower charge is extracted with a separation of 1.2 fs from the first one.

However, choosing the 3rd ionization state is arbitrary, and in order to validate the catapult scheme, a PIC simulation was completed starting with unionized Carbon atoms, and the same laser parameters as used throughout this work. As shown in Fig.  10 the laser pulse is sufficiently intense to ionize the target and form electron bunches, as before. Obviously, the electron charge distribution inside the bunch and across the target differs from when compared with Fig. 8, but this is of secondary importance.

Concluding, the catapult scheme is numerically validated through detailed PIC simulations and, when a suitable laser becomes available, this work may be used to prepare a proof-of-principle experiment.

## Supplementary Information

Supplementary Information.
