# Author Correction: TeV/m catapult acceleration of electrons in graphene layers

**DOI:** 10.1038/s41598-023-29761-z

**Published:** 2023-02-17

**Authors:** Cristian Bonţoiu, Öznur Apsimon, Egidijus Kukstas, Volodymyr Rodin, Monika Yadav, Carsten Welsch, Javier Resta-López, Alexandre Bonatto, Guoxing Xia

**Affiliations:** 1 Department of Physics, University of Liverpool, Oxford Street, Liverpool, L69 7ZE UK; 2 Cockroft Institute, Keckwick Lane, Daresbury, Warrington, WA4 4AD UK; 3 ICMUV, Instituto de Ciencia de los Materiales, Universidad de Valencia, 46071 Valencia, Spain; 4 Graduate Program in Information Technology and Healthcare Management, and the Beam Physics Group, Federal University of Health Sciences of Porto Alegre, Porto Alegre, RS 90050-170 Brazil; 5 Department of Physics and Astronomy, University of Manchester, Oxford Road, Manchester, M13 9PL UK

Correction to: *Scientific Reports* 10.1038/s41598-023-28617-w, published online 24 January 2023

The original version of this Article contained an error in the legend of Figure 1.

“Overview of the catapult electron acceleration scheme in graphene layers. Moving from left to right, as indicated by the blue arrows, a single 3 fs-long laser pulse of 100 nm wavelength and 10^21^ W/cm^2^ peak intensity, ionizes a 1.5 μm-long (y) and 1.2 μm-thick (x) stack of graphene layers. The interaction results in self-injected electrons being accelerated to ≃ 7 MeV. The image is at scale, with a 150 nm bar drawn, and for better visibility, only 15 out of 60 graphene layers are shown. The simulated normalized transverse electric field (*E*_*x*_) is shown as a surface colour plot for the same laser pulse before entering the target (left) and after leaving the target (right). This work contains 2D PIC simulations carried out in the *yx*-plane indicated in the image.”

now reads:

“Overview of the catapult electron acceleration scheme in graphene layers. Moving from left to right, as indicated by the blue arrows, a single 3 fs-long laser pulse of 100 nm wavelength and 10^21^ W/cm^2^ peak intensity, ionizes a 1.5 μm-long (y) and 1.2 μm-thick (x) stack of graphene layers. The interaction results in self-injected electrons being accelerated to ≃ 7 MeV. The image is at scale, and for better visibility, only 15 out of 60 graphene layers are shown. The simulated normalized transverse electric field (*E*_*x*_) is shown as a surface colour plot for the same laser pulse before entering the target (left) and after leaving the target (right). This work contains 2D PIC simulations carried out in the *yx*-plane indicated in the image.”

The original Article has been corrected.

