# Supplementary figures and images for: TeV/m catapult acceleration of electrons in graphene layers
Source: Sci Rep. 2023 Jan 24;13:1330. doi: 10.1038/s41598-023-28617-w (PMC9873800; doi:10.1038/s41598-023-28617-w)

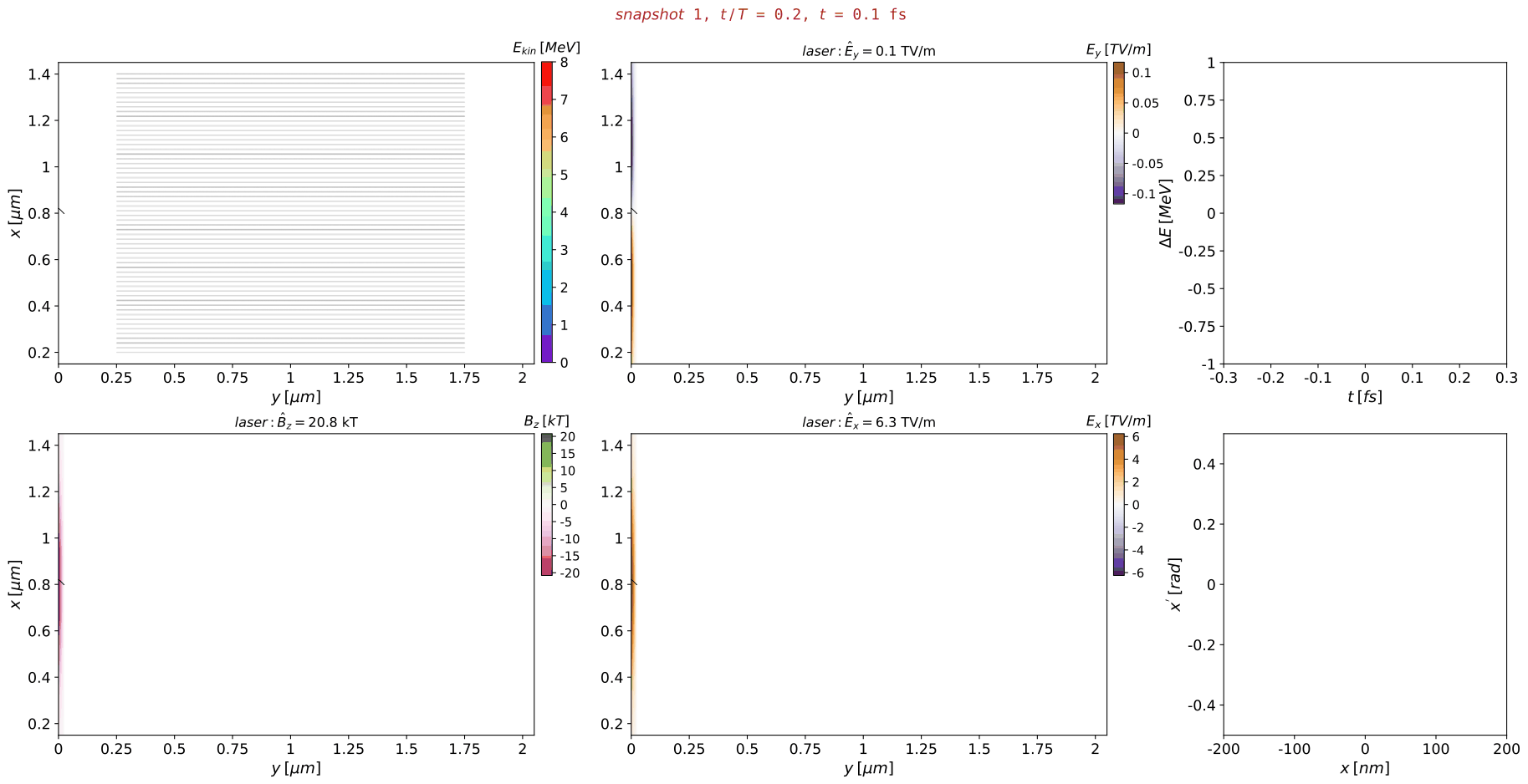

Supplement: Supplementary file 1 — Supplementary Information. [file 41598_2023_28617_MOESM1_ESM.gif]
